# Surgical Treatment of Peripartum Pelvic Ring Injury: Case Report

**DOI:** 10.1055/s-0043-1776023

**Published:** 2024-04-22

**Authors:** Ewerton Borges de Souza Lima, Marco Antonio Rojas Janco, Ubiratan Stefani de Oliveira Saturnino, Tarcísio Alves Leal, Fernando Baldy dos Reis, Luiz Fernando Cocco

**Affiliations:** 1 Escola Paulista de Medicina, Universidade Federal de São Paulo, São Paulo, SP, Brasil

**Keywords:** gestation, joint instability, pelvic bones, postpartum period, pubic symphysis, sacroiliac joint

## Abstract

During the gestational period, the pubic symphysis dilates for vaginal delivery. However, exacerbated widening may indicate ligament injury and pelvic instability, resulting in significant pain complaints. This uncommon condition is called peripartum pubic symphysis disjunction (PPSD). We herein report the case of a patient with a gestational age of 38 weeks who complained of severe pain in the lumbar and pelvic region, radiating to the right hip and knee, after vaginal delivery of a single pregnancy with no obstetric complications. The patient had extensive edema and vulvar ecchymosis, and radiographic examinations showed a pubic symphysis disjunction of 7 cm and a right sacroiliac joint opening. The literature is controversial regarding the indication of treatment for these cases, but the clinical and radiographic findings motivated the surgical treatment. We closed the pubic symphysis with orthogonal plates and stabilized the sacroiliac joint with a percutaneous screw. The patient underwent outpatient follow-up for six months, with good progression and no intercurrences, and returned to work with minimal pain, good function, and satisfied with the treatment.

## Introduction

Osteoligamentous hypermobility, especially in the pelvis, occurs during pregnancy to enable vaginal delivery. The gap in the pubic symphysis increases physiologically by about 3 mm to 5 mm. 1 A diastasis greater than 10 mm may trigger pelvic instability and pain, becoming a pathological condition called peripartum pubic symphysis disjunction (PPSD). 2

This condition is uncommon. 3 Since it is usually asymptomatic, it is difficult to calculate the number of affected patients. 4 Treatment is conservative for most cases. 2 However, we herein report the case of a patient diagnosed with severe PPSD and submitted to surgical treatment, which is rare.

## Case Report

The institutional Ethics Committee approved the present case report under CAAE number 55515122.3.0000.5505.

A 38-year-old female patient, in the 38th week of her fourth pregnancy, was admitted to the Obstetrics Department in spontaneous labor. She had been submitted to cesarean section, and denied other surgical procedures, comorbidities, or family history. The patient presented 37 cm in uterine height, 7 cm dilation, and a fetus in cephalic presentation. She progressed to vaginal labor with no need for an episiotomy or forceps, delivering a male, live neonate weighing 3,455 g, and presenting an Appearance, Pulse, Grimace, Activity, and Respiration (Apgar) score of 8/9.

In the immediate postpartum period, the patient developed lumbar and pelvic pain, which worsened when walking, and radiated to the right hip and knee, associated with loss of strength in the lower limbs and no sensitivity changes. An assessment requested by clinical medicine ruled out a potential case of deep venous thrombosis (DVT). An evaluation by neurosurgery raised the hypothesis of radicular syndrome and polyradiculoneuritis. The patient received dexamethasone 4 mg every 8 hours and underwent a computed tomography (CT) scan of the lumbar spine.

**Fig. 1:**
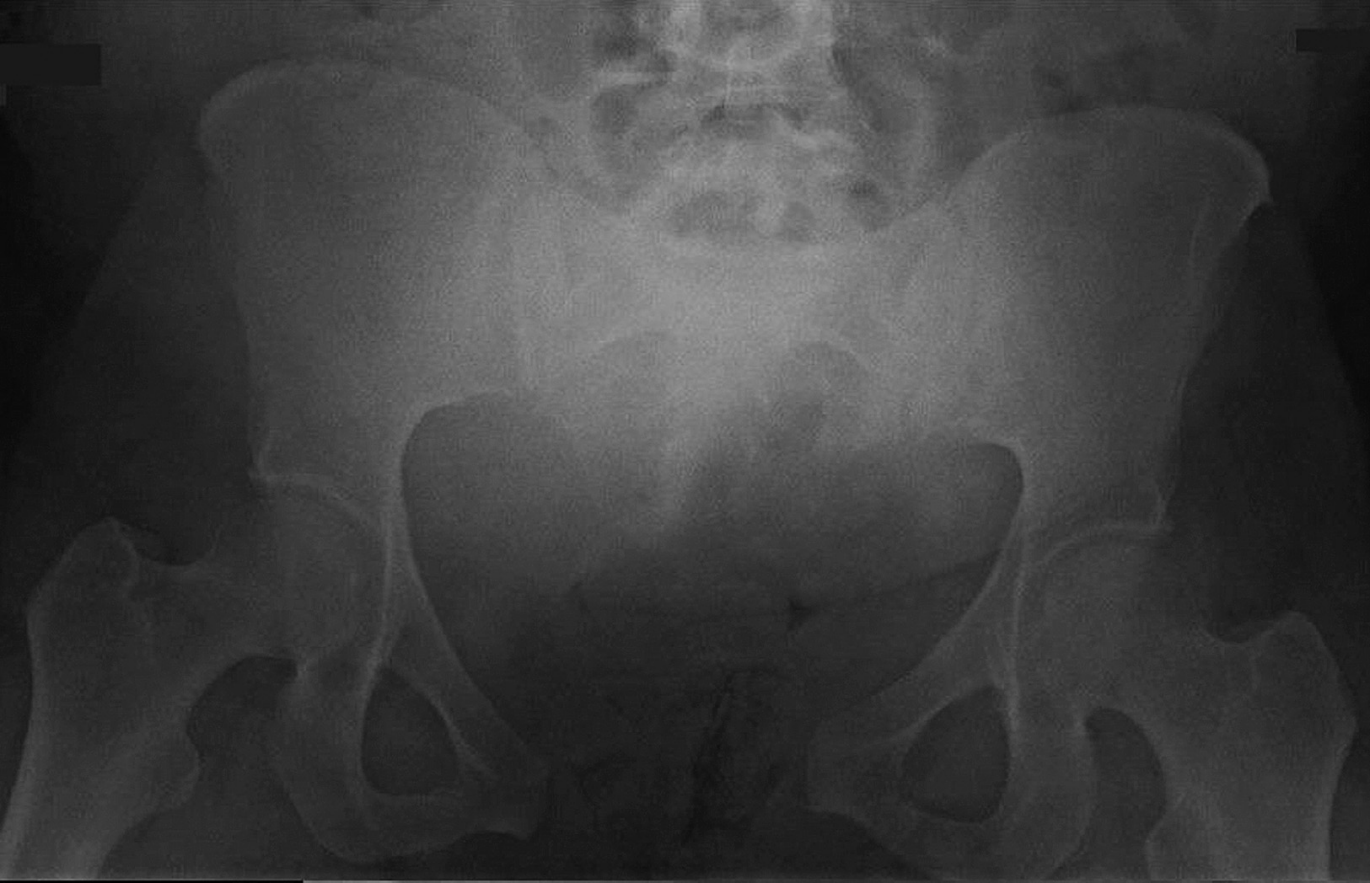
Anteroposterior radiograph of the pelvis showing pubic symphysis disjunction with 7 cm of diastasis.

**Fig. 2:**
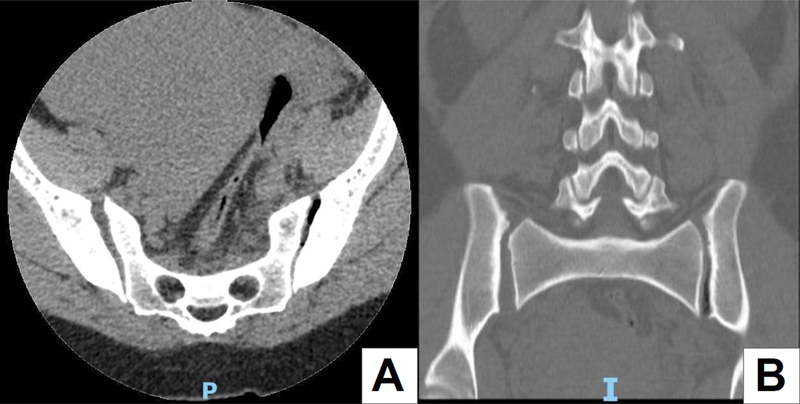
A computed tomography showing involvement of the right sacroiliac joint with a joint space of 9.7 mm compared to the contralateral joint space of 6.2 mm. ( **A** ) Axial section. ( **B** ) Coronal section.

**Fig. 3:**
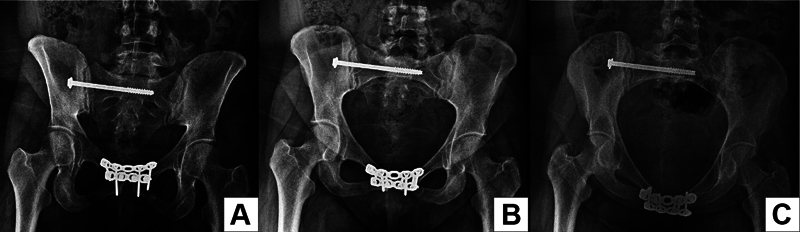
Postoperative radiographs of the reduction and fixation of the pubic symphysis and the right sacroiliac joint. ( **A** ) Anteroposterior view. ( **B** ) Inlet view. ( **C** ) Outlet view.

Two days after delivery, the patient still presented pain and ecchymosis in the vulvar region. A pelvic radiograph showed a disjunction of the pubic symphysis measuring 7 cm ( Fig. 1 ). The orthopedic team advised rest, prophylaxis for DVT, and radiographic follow-up to verify a spontaneous symphysis reduction in the puerperium. Four days after delivery, the patient remained hemodynamically stable, with a palpable gap in the pubic symphysis and no radiographic improvement. We opted for surgical treatment due to pain, especially when walking, significant pubic symphysis disjunction, and sacroiliac joint involvement on CT ( Fig. 2 ). We performed pubic symphysis reduction and fixation with two orthogonal reconstruction plates, in addition to sacroiliac joint fixation with a 7.0 mm percutaneous cannulated screw ( Fig. 3 ). The procedure was uneventful, and the patient was discharged the next day with analgesic agents, antibiotics, prophylaxis for DVT, and instructions for walking with the aid of a walker and partial weight bearing on the right lower limb.

In the second postoperative week, the patient presented with a healed surgical wound and complained of pain, with score on the Visual Analog Scale (VAS) for pain of 4 when walking, and of 2 at rest. Full weight bearing was allowed in the sixth week. Six months after surgery, the patient was already back to work as a cleaning lady, and reported being asymptomatic at rest and presenting mild pain (VAS = 2) only when climbing stairs and squatting. At the end of the treatment, the patient reported being satisfied (satisfaction of 8 out of 10).

## Discussion

In daily orthopedic and obstetric care PPSD is an uncommon condition, with an incidence ranging from 1/30,000 to 1/300 pregnancies. 3 Screening must rule out sciatica, genitourinary infection, DVT, and pubic symphysis inflammation. 5 In the case herein reported, the diagnosis of PSSD was only made after evaluation by another three medical specialties.

The patient presented classic PPSD symptoms, that is, pubic and sacral pain radiating to the lower limbs, worse when walking, which started after delivery. 5 She also presented a Destot sign (hematoma in the labia majora region), 6 which led to a radiographic examination that confirmed the condition.

Advanced maternal age and multiparity are two risk factors for PPSD observed in the case herein reported. 7 Studies have shown that nulliparity, surgical delivery, previous pelvic trauma, and epidural anesthesia are also risk factors. 2 3 7 Sung et al. 2 reported that the incidence of PPSD tends to increase per maternal age, but with no statistical significance. Although a small pelvis and a large fetus are considered risk factors, some studies have failed to show correlations. 2 8 9

Most cases of PPSD are treated conservatively with analgesic agents, rest, and pelvic brace, leading to symptom improvement within six weeks. Nevertheless, the symptoms may persist for up to six months. Radiography or ultrasound reveals stable symphysis reduction within three months. 5 In the case-control study by Sung et al., 2 all 33 cases identified underwent conservative treatment with a pelvic brace. Of these, 5 presented symphyseal diastasis greater than 4 cm, and 4 sustained pelvic pain for at least 1 year.

Some studies argue that diastases greater than 4 cm are candidates for surgical fixation. 4 In addition, sacroiliac joint involvement is frequent in symphysis diastasis greater than 2 cm. 2 Our patient presented a symphysis diastasis of 7 cm and a sacroiliac joint injury visible on CT. As such, we opted for surgery to treat the injury, prevent pelvic instability, and reduce the chance of chronic pain.

The formal indications for the surgical treatment of PPSD include chronic pain, failed attempts of symphysis closure, diastasis recurrence after pelvic belt removal, and sacroiliac instability. 5 Currently, there is a trend towards surgical treatment due to the potential for earlier walking and rehabilitation, faster recovery, and better functional outcome. Furthermore, with the conservative treatment, one-third of the patients present a chance of PPSD recurrence in subsequent vaginal deliveries. 10

The difficulty in diagnosing PPSD stands out in the present case report. It is essential to suspect this condition in patients with postpartum pelvic pain and to investigate it properly. Although the conservative treatment is preferred in most cases, signs of sacroiliac joint injury and pelvic instability may indicate surgical treatment, which yields good results in terms of pain and functional improvement and good satisfaction with the treatment.
