# Supplementary material for: Surgical Treatment of Peripartum Pelvic Ring Injury: Case Report
Source: Rev Bras Ortop (Sao Paulo). 2024 Apr 22;59(Suppl 2):e251–4. doi: 10.1055/s-0043-1776023 (PMC11679630; doi:10.1055/s-0043-1776023)
Supplement: Related texts

# Research article: Tratamento cirúrgico de lesão periparto do anel pélvico: Relato de caso

**Keywords:** articulação sacroilíaca, gestação, instabilidade articular, ossos pélvicos, período pós-parto, sínfise pubiana

## Abstract

Durante o período gestacional, ocorre a dilatação da sínfise púbica, de forma a permitir o parto vaginal. No entanto, o alargamento exacerbado pode indicar lesão ligamentar, instabilidade pélvica, e desencadear queixa álgica importante. Tal quadro é uma condição incomum, denominada disjunção da sínfise púbica periparto (DSPP). Neste artigo, relatamos o caso de uma paciente com idade gestacional de 38 semanas que mantinha queixa álgica intensa na região lombar e pélvica, irradiada para o quadril e o joelho direito, após parto normal de gestação única, sem intercorrências obstétricas. A paciente apresentava extenso edema e equimose vulvar, e exames radiográficos evidenciaram disjunção da sínfise púbica de 7 cm e abertura da articulação sacroilíaca direita. Há controvérsias na literatura quanto à indicação do tratamento desses casos, mas os achados clínicos e radiográficos motivaram a opção pelo tratamento cirúrgico. Realizou-se o fechamento da sínfise púbica com placas ortogonais, além da estabilização da articulação sacroilíaca com parafuso percutâneo. A paciente fez acompanhamento ambulatorial por seis meses, com boa evolução e sem intercorrências, e retornou às atividades laborais com dor mínima, boa função e satisfação com o tratamento.

## Introdução

Na gestação, ocorre o fenômeno de hipermobilidade osteoligamentar, em especial da pelve, para permitir o parto vaginal. O espaço da sínfise púbica aumenta fisiologicamente cerca de 3 mm a 5 mm. 1 Se a diástase atingir mais de 10 mm, pode desencadear instabilidade pélvica e dor, e se torna uma condição patológica denominada disjunção da sínfise púbica periparto (DSPP). 2

Trata-se de uma lesão incomum 3 e, pelo fato de geralmente ser assintomática, é difícil o cálculo exato dos pacientes acometidos. 4 A maioria dos casos é tratada de maneira conservadora; 2 no entanto, neste artigo, relatamos o caso de uma paciente diagnosticada com DSPP grave para a qual se indicou o tratamento cirúrgico, uma situação rara.

## Relato de Caso

Este relato de caso foi aprovado pelo Comitê de Ética de nossa instituição sob o número CAAE: 55515122.3.0000.5505.

Uma paciente do sexo feminino, de 38 anos, na 38ª semana de sua quarta gestação, foi admitida no Setor de Obstetrícia em trabalho de parto espontâneo. Ela já havia sido submetida a parto por cesárea, negava outros procedimentos cirúrgicos, comorbidades ou antecedentes familiares, e apresentava altura uterina de 37 cm, dilatação de 7 cm, e feto em apresentação cefálica. A paciente evoluiu para trabalho de parto vaginal, sem necessidade de episiotomia ou fórceps, e deu à luz um neonato vivo, do sexo masculino, que pesava 3,455 g, e tinha pontuação de 8/9 na escala de Aparência, Pulso, Gesticulação, Atividade, Respiração (Apgar).

No pós-parto imediato, a paciente evoluiu com dor em região lombar e pélvica, pior à deambulação, irradiada para o quadril e o joelho direito, associada a perda de força nos membros inferiores, sem alteração de sensibilidade. Foi solicitada avaliação da clínica médica, que descartou hipótese de trombose venosa profunda (TVP), e da neurocirurgia, que levantou as hipóteses de síndrome radicular e polirradiculoneurite. Prescreveu-se dexametasona 4 mg de 8/8h e solicitou-se tomografia computadorizada de coluna lombar.

Dois dias após o parto, a paciente manteve o quadro álgico, e observou-se equimose na região vulvar. Uma radiografia de pelve evidenciou disjunção da sínfise púbica de 7 cm ( Fig. 1 ). A equipe ortopédica orientou repouso, profilaxia para TVP e acompanhamento radiográfico para verificar redução espontânea da sínfise no puerpério. Quatro dias após o parto, a paciente mantinha-se estável hemodinamicamente, com disjunção palpável na sínfise púbica e sem melhora radiográfica. Devido à manutenção do quadro álgico, principalmente à deambulação, e à disjunção importante da sínfise púbica e ao acometimento da articulação sacroilíaca, verificados na tomografia ( Fig. 2 ), optou-se pelo tratamento cirúrgico. Realizou-se redução da sínfise púbica e fixação com duas placas de reconstrução ortogonais, além da fixação da articulação sacroilíaca com parafuso canulado de 7.0 mm de fixação percutânea ( Fig. 3 ). O procedimento foi realizado sem intercorrências, e a paciente teve alta hospitalar no dia seguinte, com analgésicos, antibiótico, profilaxia para TVP e orientação de deambulação com auxílio de andador e carga parcial no membro inferior direito.

**Fig. 1:** Radiografia anteroposterior da pelve que evidencia disjunção da sínfise púbica com 7 cm de diástase.

**Fig. 2:** Tomografia computadorizada que evidencia acomentimento da articulação sacroilíaca direita, com espaço articular de 9,7 mm em comparação a 6,2 mm do lado oposto. ( **A** ) Corte axial. ( **B** ) Corte coronal.

**Fig. 3:** Radiografias pós-operatórias da redução e fixação da sínfise púbica e da articulação sacroilíaca direita. ( **A** ) Incidência anteroposterior. ( **B** ) Incidência *inlet* . ( **C** ) Incidência *outlet* .

Na segunda semana de pós-operatório, a paciente apresentava-se com ferida operatória cicatrizada, queixa álgica, com pontuação de 4 na Escala Visual Analógica (EVA) de dor ao andar e, em repouso, pontuação de 2. Ela iniciou tratamento fisioterápico e apresentou boa evolução. Carga total foi liberada na sexta semana e, no sexto mês de pós-operatório, a paciente já havia retornado ao trabalho (faxineira), assintomática quando em repouso e mencionava queixa álgica discreta (EVA = 2) apenas ao subir escadas e ao se agachar. Ao final do tratamento, a paciente afirmou estar satisfeita (satisfação 8 de 10).

## Discussão

A DSPP é uma condição incomum no cotidiano ortopédico e obstétrico, com incidência que varia de 1/30.000 a 1/300 gestações. 3 A investigação deve excluir ciatalgia, infecção geniturinária, TVP e inflamação da sínfise púbica. 5 No caso aqui relatado, o diagnóstico de DSPP só foi realizado depois da avaliação de outras três especialidades médicas.

A paciente apresentava sintomas clássicos para DSPP: dor púbica e sacral irradiada para os membros inferiores, pior à deambulação, iniciada após o parto. 5 Apresentou também sinal de Destot (hematoma na região dos grandes lábios), 6 o que levou à realização da radiografia que evidenciou a lesão.

Idade materna avançada e multiparidade são dois fatores de risco para DSPP presentes neste caso. 7 Estudos demonstraram que nuliparidade, parto cirúrgico, trauma pélvico prévio e anestesia peridural também são fatores de risco. 2 3 7 No estudo de Sung et al. 2 a incidência de DSPP tendeu a aumentar com o aumento da idade materna, mas não foi estatisticamente significativa. Pelve pequena e feto grande são considerados fatores de risco, mas alguns estudos demonstraram não haver correlação. 2 8 9

A maioria dos casos de DSPP é tratada conservadoramente com analgésicos, repouso e colete pélvico, e apresenta melhora em seis semanas, mas os sintomas podem perdurar por até seis meses. A redução estável da sínfise é observada na radiografia ou na ultrassonografia dentro de três meses. 5 No estudo de caso-controle de Sung et al., 2 todos os 33 casos identificados foram tratados de forma conservadora com colete pélvico. Destes, 5 apresentaram diástase da sínfise maior do que 4 cm, sendo que 4 mantiveram dor pélvica por pelo menos 1 ano.

Alguns estudos discutem que diástases maiores do que 4 cm são candidatas à fixação cirúrgica. 4 Além disso, quando a diástase da sínfise é maior do que 2 cm, o acometimento da articulação sacroilíaca é frequente. 2 Nossa paciente apresentava diástase da sínfise de 7 cm, além de lesão da articulação sacroilíaca visível na tomografia computadorizada. Por esses motivos, optou-se pelo tratamento cirúrgico, para tratar a lesão, prevenir a instabilidade pélvica e diminuir a chance de dor crônica.

São indicações formais de tratamento cirúrgico para DSPP: dor crônica, falha de fechamento da sínfise, recorrência da diástase após retirada do cinto pélvico e instabilidade sacroilíaca. 5 Atualmente, há uma tendência ao tratamento cirúrgico devido à possibilidade de marcha e reabilitação precoce, recuperação mais rápida e melhor desfecho funcional. Além disso, com o tratamento conservador, um terço das mulheres tem chance de recorrência de DSPP nos partos vaginais subsequentes. 10

Destaca-se no relato de caso a dificuldade para o diagnóstico da DSPP. É essencial que haja a suspeita dessa condição em quadros de dor pélvica pós-parto e que seja realizada investigação. O tratamento conservador é o de escolha na maioria dos casos, porém, quando há indícios de lesão da articulação sacroilíaca e instabilidade pélvica, o tratamento cirúrgico pode ser indicado, com bons resultados em termos de melhora da dor e da função, além de boa satisfação com o tratamento.
